# Lexical Effects on the Perceived Clarity of Noise-Vocoded Speech in Younger and Older Listeners

**DOI:** 10.3389/fpsyg.2022.837644

**Published:** 2022-04-01

**Authors:** Terrin N. Tamati, Victoria A. Sevich, Emily M. Clausing, Aaron C. Moberly

**Affiliations:** ^1^Department of Otolaryngology – Head and Neck Surgery, The Ohio State University Wexner Medical Center, Columbus, OH, United States; ^2^Department of Otorhinolaryngology/Head and Neck Surgery, University Medical Center Groningen, University of Groningen, Groningen, Netherlands; ^3^Department of Speech and Hearing Science, The Ohio State University, Columbus, OH, United States

**Keywords:** speech clarity, noise-vocoded speech, priming, lexical properties, aging

## Abstract

When listening to degraded speech, such as speech delivered by a cochlear implant (CI), listeners make use of top-down linguistic knowledge to facilitate speech recognition. Lexical knowledge supports speech recognition and enhances the perceived clarity of speech. Yet, the extent to which lexical knowledge can be used to effectively compensate for degraded input may depend on the degree of degradation and the listener’s age. The current study investigated lexical effects in the compensation for speech that was degraded via noise-vocoding in younger and older listeners. In an online experiment, younger and older normal-hearing (NH) listeners rated the clarity of noise-vocoded sentences on a scale from 1 (“very unclear”) to 7 (“completely clear”). Lexical information was provided by matching text primes and the lexical content of the target utterance. Half of the sentences were preceded by a matching text prime, while half were preceded by a non-matching prime. Each sentence also consisted of three key words of high or low lexical frequency and neighborhood density. Sentences were processed to simulate CI hearing, using an eight-channel noise vocoder with varying filter slopes. Results showed that lexical information impacted the perceived clarity of noise-vocoded speech. Noise-vocoded speech was perceived as clearer when preceded by a matching prime, and when sentences included key words with high lexical frequency and low neighborhood density. However, the strength of the lexical effects depended on the level of degradation. Matching text primes had a greater impact for speech with poorer spectral resolution, but lexical content had a smaller impact for speech with poorer spectral resolution. Finally, lexical information appeared to benefit both younger and older listeners. Findings demonstrate that lexical knowledge can be employed by younger and older listeners in cognitive compensation during the processing of noise-vocoded speech. However, lexical content may not be as reliable when the signal is highly degraded. Clinical implications are that for adult CI users, lexical knowledge might be used to compensate for the degraded speech signal, regardless of age, but some CI users may be hindered by a relatively poor signal.

## Introduction

An important and distinctive property of speech perception is its robustness in the face of a wide range of adverse and challenging conditions. Successful recognition of a spoken word involves rapid mapping of the acoustic signal onto lexical representations stored in long-term memory (e.g., McClelland and Elman, 1986; Norris, 1994; Luce and Pisoni, 1998). In favorable listening conditions, lexical access occurs rapidly and automatically, with minimal recruitment of cognitive processing to disambiguate the message. In everyday, real-world environments, however, the speech signal is often distorted by environmental degradations, such as background noise or competing speech, as well as source degradations from variability arising from talkers with different developmental, social, and language histories (e.g., Mattys et al., 2012; Gilbert et al., 2013). Further, hearing-impaired listeners must also cope with additional degradations due to reduced audibility and/or distortions specific to their type, degree, and configuration of hearing loss. Even rehabilitative devices, such as hearing aids or cochlear implants (CIs), can preserve or introduce spectral degradations, despite partially restoring audibility. As a result of these combined sources of adversity, speech recognition in real-world conditions is challenging (e.g., Johnson et al., 2016; Meister et al., 2016; Hughes et al., 2018; Janse and Andringa, 2021), and resolving the increased ambiguity arising from these adverse factors requires the recruitment of cognitive mechanisms, such as attention, semantic and syntactic constraints, and lexical knowledge (e.g., Pichora-Fuller, 2008; Başkent et al., 2016a; Koeritzer et al., 2018). The effective use of cognitive processes and linguistic knowledge to recognize degraded speech likely depends on both bottom-up signal quality and the top-down cognitive-linguistic skills of the individual listener (e.g., Rönnberg et al., 2013; Başkent et al., 2016a; Moberly et al., 2021). Still, it is relatively unclear how these bottom-up and top-down processes interact to impact speech recognition, and further how the contribution of these factors may depend on the age of the listener. The current study explores the contribution of bottom-up and top-down factors – and their interaction – to the perceived clarity of noise-vocoded speech in younger and older adults with normal hearing (NH).

### Recognition of Degraded Speech

Top-down mechanisms are especially relevant for hearing impaired adults with CIs. Adult CI users must achieve successful daily communication relying on speech signals that are heavily reduced in acoustic-phonetic detail compared to what is typically available to NH listeners, due to the limitations of the electrode-nerve interface and relatively broad electrical stimulation of the auditory nerve (for a review, see Başkent et al. (2016b)). This reduced spectral resolution limits the accurate recognition of speech in CI users (Henry et al., 2005). CI users may achieve accurate recognition of the degraded speech delivered by the device, but do so by relying on predictive coding and downstream cognitive resources (e.g., Pals et al., 2013; Bhargava et al., 2014; Winn et al., 2015; Başkent et al., 2016a). However, individual CI users display variability in spectral resolution across the electrode array (Won et al., 2007), which may be related to auditory nerve health, electrode placement, or other device or surgical factors (e.g., Blamey et al., 2013; Başkent et al., 2016b). Poorer spectral resolution in CI users may contribute to increased difficulty in recognizing speech (Henry et al., 2005; Won et al., 2007; Moberly et al., 2018b) and impact the ability to effectively use top-down resources (Bhargava et al., 2014; Pals et al., 2020).

Increased signal degradation may result in greater relative reliance on top-down cognitive-linguistic resources. For example, Pals et al. (2013) examined listening effort in the recognition of noise-vocoded speech. Noise-vocoding is commonly used to simulate – albeit imperfectly – the signal delivered by a CI and to introduce varying degrees of spectral degradation experimentally. In their study, increasing spectral resolution in the noise-vocoder simulations of CI hearing resulted in reduced response times in a dual-task paradigm, suggesting that listening effort decreases with increased signal quality. In a later study, Pals et al. (2020) examined the effect of the number of spectral channels (i.e., spectral resolution) on speech comprehension and listening effort in CI users. They found that increasing the number of spectral channels leads to an improvement in speech comprehension and response times in the sentence verification task, suggesting increased signal quality improves speech comprehension and listening effort. Interestingly, this effect was not observed in the dual-task paradigm, which the authors interpreted as evidence that changes in listening effort as a function of signal degradation may not be well reflected in tasks assessing speech recognition accuracy. Similarly, conventional measures of speech recognition accuracy may not be as sensitive to subtle differences in signal degradation and listening effort compared to measures that capture the time course and processes underlying speech perception and spoken word recognition (e.g., Başkent et al., 2016a; Pisoni et al., 2017; Moberly et al., 2018a; Winn and Teece, 2021). Measures involving subjective assessment of speech clarity may also be more sensitive to differences in signal quality since they would allow the listener to make more subtle distinctions between degraded signals (e.g., Sohoglu et al., 2014), even when using a wide range of degrees of degradation that may produce ceiling and/or floor effects in a word or sentence recognition task.

### Lexical Knowledge in Degraded Speech Perception

To cope with degraded speech, listeners utilize several linguistic resources, including semantic context, syntactic structure, and lexical information (e.g., Pichora-Fuller, 2008; Başkent et al., 2016a; Wagner et al., 2016; Koeritzer et al., 2018). Regarding lexical information, listeners make use of linguistic context providing the lexical and phonological form of an utterance to make predictions about its content. The perceptual processing of speech is facilitated when a listener is provided with text that partially or completely matches the target utterance prior to its auditory presentation (e.g., Goldinger et al., 1992; Buchwald et al., 2009; Chng et al., 2019). Form-based prediction from exact matching text provides specific information about the lexical and phonological content of an upcoming utterance and allows for the activation of the lexical items in that utterance. In this manner, top-down lexical and phonological information provided visually by matching text primes enhances the perception of noise-vocoded speech (Davis et al., 2005; Hervais-Adelman et al., 2011; Wild et al., 2012; Signoret et al., 2018; Signoret and Rudner, 2019). Recently, Signoret et al. (2018) used a speech clarity rating task to assess the effects of bottom-up spectral resolution from acoustic noise-vocoding and top-down form-based prediction from matching text primes as well as meaning-based prediction from supportive semantic context on the perceived clarity of degraded speech in NH young to middle aged adults. The authors found that speech clarity ratings were sensitive to differences in the spectral resolution of the noise-vocoded speech (manipulated in that study by the number of vocoder channels). Moreover, they found evidence for independent and additive effects of form- and meaning-based prediction on clarity ratings. Together, these previous studies also demonstrate that a speech clarity rating task may be a sensitive and useful tool for assessing top-down effects on the perception of degraded speech.

The lexical properties of the words within an utterance also influence the speed and accuracy of spoken word recognition (e.g., Luce and Pisoni, 1998). According to most accounts, spoken word recognition involves the activation of a set of candidate words including the target and words that are phonologically-similar to the target. Words that differ from the target word by a single phoneme that is substituted, deleted, or added are considered to share phonological similarity and form part of the target word’s phonological neighborhood (Luce and Pisoni, 1998). As more information becomes available, the target word is selected from the candidate words, while competitors must be inhibited (e.g., Marslen-Wilson, 1987; Luce and Pisoni, 1998). Two lexical properties – lexical frequency (i.e., frequency of occurrence in a spoken language) and neighborhood density (i.e., number of phonologically-similar lexical neighbors) – play key roles in the discrimination and selection of the target item. Words with higher lexical frequency and fewer neighbors (“easy” words) are easier to recognize than words with lower lexical frequency and more neighbors (“hard” words) since there is greater activation of the target word and less competition from neighbors. Accordingly, easy words have consistently been found to be more quickly and accurately recognized than hard words for NH listeners, particularly in the presence of noise or other sources of adversity (e.g., Howes, 1957; Savin, 1963; Sommers et al., 1997; Bradlow and Pisoni, 1999; Taler et al., 2010). Effects of lexical frequency and neighborhood density have also been observed for NH listeners with noise-vocoded speech (Tamati et al., 2020b) and CI users (Tamati and Moberly, 2021). Thus, the lexical content of an utterance may be a source of top-down compensatory information that has a relatively strong impact on the recognition of degraded speech.

### Interactions of Bottom-Up and Top-Down Processing

Relative reliance on top-down compensatory mechanisms in speech understanding may depend on the degree of degradation of the speech signal. Listeners rely more on top-down mechanisms to a certain degree when speech is degraded by noise or other sources of adversity (e.g., Kalikow et al., 1977; Luce and Pisoni, 1998; Vitevitch and Luce, 1999; Mattys et al., 2012). However, reliance on top-down processing may decrease when the degree of degradation of the speech signal is more extreme (Boothroyd and Nittrouer, 1988; Mattys et al., 2009; Clopper, 2012; Bhargava et al., 2014; Gelfand et al., 2014). Linguistic information conveyed by a severely degraded signal may be undetectable or misleading (Samuel, 1981; Król and El-Deredy, 2011; Bhargava et al., 2014; Sohoglu et al., 2014), resulting in reduced reliance on higher-level linguistic knowledge and greater reliance on lower-level segmental cues. As such, the speech signal must provide sufficient acoustic-phonetic detail to support higher-level processing (Aydelott and Bates, 2004; Mattys et al., 2005, 2009; Clopper, 2012). Interestingly, in the study by Signoret et al. (2018) described above, the authors observed that form- (matching text primes) and meaning-based prediction (semantic context) had greater effects for more degraded signals compared to more favorable signals. In a follow-up study, Signoret and Rudner (2019) also found evidence for the interaction between top-down and bottom-up processes in speech clarity ratings in a group of older, hearing-impaired adults. With less degraded speech, older, hearing-impaired listeners benefited from semantic context. However, with more degraded speech, the benefit from semantic context was observed only when matching text primes preceded the sentence. Further, unlike findings in the original Signoret et al. (2018) study of younger listeners, benefits from form- and meaning-based prediction were not related to working memory capacity in the older, hearing-impaired listeners, suggesting that they may have exceeded their available resources to effectively process the degraded speech.

Similarly, findings from previous studies examining variability in speech recognition outcomes in adult CI users demonstrate that some CI users may be able to more effectively use top-down compensation (Bhargava et al., 2014; Moberly et al., 2014, 2016; Başkent et al., 2016a). Relatively poorer performing CI users have demonstrated a reduced ability to take advantage of top-down compensatory mechanisms (e.g., Liu et al., 2004; Bhargava et al., 2014; Başkent et al., 2016a), suggesting a reduced role of cognitive-linguistic abilities for poorer performers. Additionally, Moberly et al. (2021) found that the contribution of cognitive-linguistic abilities to speech recognition outcomes in adult CI users depended on individual bottom-up auditory sensitivity. Cognitive-linguistic abilities contributed less to speech recognition outcomes for adult CI users with poor auditory spectro-temporal resolution compared to CI users with better auditory resolution. Similarly, specifically comparing performance between groups of CI users with the poorest and best outcomes, Tamati et al. (2020a) suggested that top-down processes may play a limited role in speech recognition in CI users with the poorest bottom-up auditory sensitivity. However, although many adult CI users are typically of advanced age, these studies did not consider how aging may have contributed to individual differences in top-down compensation. Thus, for individual adult CI users, the ability to use top-down compensatory mechanisms to recognize the degraded signal delivered by a CI depends on cognitive-linguistic ability and, crucially, on the quality of the signal processed by the implant and delivered to the auditory cortex. Yet, it is still unknown how aging may alter the use of top-down compensatory strategies for degraded speech understanding.

### The Effects of Aging on Top-Down Compensation

Top-down compensation for degraded speech among older adults may be impacted by age-related declines in neurocognitive functioning and auditory sensitivity. Older adults with “age-normal” hearing (i.e., normal or near-normal thresholds to tones on audiometric testing) demonstrate poorer spectro-temporal processing of auditory input (Fitzgibbons and Gordon-Salant, 1994; Schmiedt, 2010; Tun et al., 2012), as well as aging-related declines in neurocognitive functions of working memory capacity, inhibition-concentration, information-processing speed, and non-verbal reasoning (i.e., fluid intelligence). These age-related declines in top-down cognitive functioning and bottom-up auditory processes may contribute to overall poorer speech recognition abilities compared to younger adults (Pichora-Fuller and Singh, 2006; Arehart et al., 2013). Further, older listeners may be even more greatly impacted by adverse conditions, such as speech degraded by vocoding (Rosemann et al., 2017; Moberly et al., 2018b).

Some processes that may help support the perception of degraded speech are fortunately maintained during aging. Specifically, older listeners may rely upon prior knowledge (i.e., crystallized intelligence – knowledge previously acquired through prior learning and experiences, such as vocabulary knowledge) to enhance the processing of degraded speech. In contrast with fluid intelligence, crystallized intelligence is typically maintained in older age (Salthouse, 1993; Wingfield et al., 1994; Ryan et al., 2000; Park et al., 2002). Previous findings suggest that older adults may take advantage of crystallized intelligence in adverse listening conditions to the same extent – or possibly even more so – than younger listeners (e.g., Balota and Duchek, 1991; Wingfield et al., 1994; Pichora-Fuller et al., 1995; Valencia-Laver and Light, 2000; Daneman et al., 2006; Sheldon et al., 2008). Top-down compensation in older adults may therefore specifically involve reliance on linguistic knowledge, such as through use of supportive semantic or syntactic context (e.g., Pichora-Fuller, 2008) and lexical information (e.g., Schneider et al., 2016), during the recognition of degraded speech.

For older listeners, lexical knowledge may play an important role in the processing of degraded speech. Some previous studies suggest that older adults may benefit at least as much, if not more, as younger adults from exact or partially matching auditory or text primes (e.g., Wu et al., 2012; Getzmann et al., 2014; Freyman et al., 2017; Ouyang et al., 2020). Differences among older and younger listeners may arise from changes in lexical processing due to age-related declines in the top-down processing of speech (e.g., Federmeier et al., 2003) as well as increases in or maintenance of vocabulary knowledge (Verhaeghen, 2003) across the lifespan. Previous studies examining lexical competition in speech recognition suggest that older listeners display difficulty in resolving lexical competition during speech recognition (Sommers, 1996; Sommers and Danielson, 1999; Helfner and Jesse, 2015), potentially due to age-related declines in inhibitory control as well as increases in vocabulary size. Older adults show less accurate recognition of words with high neighborhood density in noise compared to younger listeners (Sommers, 1996; Sommers and Danielson, 1999). Examining the effects of lexical competition on word-in-sentence recognition, Taler et al. (2010) found that difference scores between accuracy for words with high and low neighborhood density in challenging conditions (lower SNR of −3 dB) were negatively related to inhibitory control across younger and older listeners, demonstrating that those with stronger inhibitory control were less affected by density effects. Further, the recognition of words with high neighborhood density, but not words with low neighborhood density, relates to stronger inhibitory control (Green and Barber, 1981, 1983; Jerger et al., 1993). Finally, increases in vocabulary size in aging may result in increased lexical competition in older adults (e.g., Salthouse, 2004; McAuliffe et al., 2013; Ramscar et al., 2014; Carroll et al., 2016). Thus, age-related changes in the top-down processing of speech may result in decreased lexical discriminability for words with many phonologically-similar neighbors.

Age-related changes in the use of lexical frequency information may also contribute to difficulties in resolving lexical competition during speech recognition. Results from Taler et al. (2010) suggest that lexical frequency effects on word-in-sentence recognition are similar across the lifespan. In that study, both older and younger listeners responded more accurately and quickly to sentences containing high-frequency words than low-frequency words. However, studies using other approaches suggest that older adults rely more heavily on lexical frequency than younger adults. Older adults appear to show increased activation of high frequency target words (and competitors) and less competition from low frequency competitors (Revill and Spieler, 2012). In an eye-tracking study, Revill and Spieler (2012) found that older adults were more likely to fixate high-frequency phonological competitors compared to younger listeners when listening to speech degraded with white noise; in contrast, younger adults were not more likely to fixate high-frequency competitors. Similarly, results in visual word processing demonstrate that older readers show stronger effects of word frequency than younger readers (Spieler and Balota, 2000; Balota et al., 2004). Together, these findings suggest that lexical effects (originating from matching text primes and/or the lexical content of the target stimulus) may have a greater impact on speech processing in older listeners.

### The Current Study

The current study investigated the top-down cognitive-linguistic and bottom-up sensory factors that affect the perceived clarity of speech in NH younger and older adults using an online speech clarity rating task. Speech was degraded using acoustic noise-vocoder simulations of CI hearing. The use of simulations allows for the signal parameters to be well controlled in order ensure that NH listeners experience similar degrees of signal degradation. Additionally, the linguistic and hearing histories of NH listeners can be better controlled, in contrast with typical adult CI users who vary in age, durations of deafness, length of CI use, and etiology of hearing loss, which may influence overall speech recognition abilities (e.g., Blamey et al., 2013). Greater control over these factors facilitates the evaluation of how bottom-up and top-down processing impacts speech recognition. Finally, findings using noise-vocoded speech have potential clinical relevance for understanding speech recognition outcomes in CI users, providing valuable insight into how spectral degradation affects speech recognition outcomes (e.g., Friesen et al., 2001).

The main goal of the current study was to investigate the top-down cognitive-linguistic factors that affect the perceived clarity of noise-vocoded speech, and how these factors may interact with bottom-up sensory factors. Given that the current study was administered online, our first goal was to determine if speech clarity ratings provided within an online experimental procedure would be consistent with previous findings obtained with in-person experimental procedures. In line with previous studies (Signoret et al., 2018; Signoret and Rudner, 2019), we sought to evaluate if online speech clarity ratings for 8-channel acoustic noise-vocoder simulations of CI hearing are sensitive to signal quality differences. To investigate the effect of spectral resolution on speech clarity, the current study manipulated the sharpness of the slope of the bandpass filters to simulate current spread in the cochlea. The amount of spread of excitation in the cochlea determines the extent to which individual stimulation channels of the implant interact (e.g., Black and Clark, 1980; Bingabr et al., 2008; Gaudrain and Başkent, 2015; Koelewijn et al., 2021). Three vocoder conditions were included to simulate low spread (LS), medium spread (MS), and high spread (HS) of excitation (and decreasing spectral resolution, respectively), in order to obtain varying degrees of degradation. In prior studies, simulating electrical current spread in the cochlea by systematically varying synthesis filter slopes has yielded a wide range of performance on speech recognition accuracy in NH listeners (Bingabr et al., 2008; Oxenham and Kreft, 2014; Winn et al., 2016; Mehta et al., 2020). For example, Bingabr et al. (2008) found that listeners achieved more accurate recognition of 8-channel vocoded words with steeper filter slopes (lower spread, higher spectral resolution): accuracy increased from about 40–80% as filter slopes increased incrementally from 14 dB/octave (lowest spectral resolution) to 110 dB/octave (highest spectral resolution). Since more intelligible speech is correlated with higher ratings of speech clarity (Eisenberg et al., 1998), we similarly expected to find increasing ratings of speech clarity as we increased synthesis filter slopes (i.e., provided more favorable spectral resolution). If the online speech clarity ratings are consistent with previous in-person results, increasing spectral resolution would be expected to result in higher perceived clarity (i.e., LS < MS < HS).

Second, we examined the effects of form-based text priming and the effects of lexical content (lexical frequency and neighborhood density) on the perceived clarity of noise-vocoded speech. To do so, we first attempted to replicate the effect of matching text primes observed in previous studies by Signoret et al. (2018) and Signoret and Rudner (2019) within the online experimental procedure. Text primes that were either matching (i.e., the text prime and the target sentence were the same) or non-matching (i.e., the prime and the target sentence were different) were presented prior to a target sentence. Consistent with the findings from these previous studies, we expected that matching primes would enhance the perceived clarity of vocoded target sentences, compared to non-matching primes.

Expanding on the earlier findings, we also examined the effects of lexical frequency and neighborhood density on the perceived clarity of noise-vocoded sentences. Sentences used in the current study were from the Veteran’s Affairs Sentence Test (VAST; Bell and Wilson, 2001), which was developed to control for frequency of word use and lexical confusability, based on Luce and Pisoni (1998). Each VAST sentence contained key words that had relatively (1) high or low lexical frequency and (2) high or low neighborhood density, resulting in four sentence types: high lexical frequency, low neighborhood density (HL); high lexical frequency, high neighborhood density (HH); low lexical frequency, low neighborhood density (LL); and low lexical frequency, high neighborhood density (LH). Based on previous findings regarding the effects of lexical frequency and neighborhood density in the recognition of noise-vocoded speech or in CI users (e.g., Tamati et al., 2020b; Tamati and Moberly, 2021), we expected that the lexical characteristics of the key words of a sentence would determine its perceived clarity. That is, we expected that sentences with high frequency key words would be perceived as clearer than sentences with low frequency key words, and that sentences with key words with low neighborhood density would be perceived as clearer than sentences with key words with high neighborhood density.

We further predicted an interaction between bottom-up signal quality (i.e., vocoder condition) and top-down cognitive-linguistic factors (i.e., priming and lexical content). Previous findings suggest decreased reliance on top-down processing under conditions of severe spectro-temporal degradation (e.g., Bhargava et al., 2014; Başkent et al., 2016a; Moberly et al., 2021). In the current study, vocoder conditions were designed to simulate decreasing degrees of spectral resolution. If top-down processing contributes less when the signal is more severely degraded, then lexical knowledge would be expected to contribute less to the perceived clarity of sentences with relatively poor signal quality (HS), and contribute relatively more for sentences with relatively more favorable signal quality (MS and LS). That is, lexical information should demonstrate a relatively stronger effect on perceived speech clarity in the MS and LS conditions compared to the HS condition (i.e., larger differences between priming conditions and sentence types). However, the two sources of lexical information in the current study (i.e., matching text primes presented visually and lexical content presented auditorily) differ by their susceptibility to signal degradation; as such, they may differ in their contributions under severely degraded conditions.

Finally, the current study sought to assess the impact of aging on how top-down cognitive-linguistic factors contribute to the perceived clarity of noise-vocoded speech. Previous research from Signoret et al. (2018) and Signoret and Rudner (2019) found potential differences in the interaction of top-down and bottom-up processes in younger versus older, hearing-impaired adults, the latter of whom seemed to exhibit less top-down compensation with degrees of degradation at which the younger adults had shown top-down effects. The findings of the two studies demonstrating reduced top-down compensation in older, hearing-impaired adults under conditions of more severe degradation suggest that there may be group differences in top-down compensation attributable to hearing impairment and/or age. Yet, because these two factors were conflated in the second study, it is unclear if aging alone impacts top-down processing and its interactions with signal quality. Based on previous findings on lexical effects on speech recognition across the lifespan, it was expected that older adults would be able to utilize top-down lexical knowledge to the same extent as, if not more than, younger adults, at least in conditions of more favorable signal quality. However, if the use of top-down lexical knowledge is restricted by poorer auditory and/or cognitive functioning, then older adults would not demonstrate the benefits from matching text primes and lexical content in conditions of poorer signal quality. Moreover, if poorer neurocognitive functioning contributes to this deficiency, then older adults may be limited in their use of lexical information, regardless of whether that information is delivered visually (i.e., matching text primes) or auditorily (i.e., lexical content). In contrast, if poorer auditory sensitivity contributes to this deficiency, then older adults would be expected to demonstrate less effective use of lexical knowledge in the perceived clarity of noise-vocoded speech specifically when relying exclusively on auditory information, thereby resulting in a relatively smaller effect of lexical content.

To summarize, this study tested the following hypotheses. First, decreasing spectral resolution would lead to lower perceived clarity for noise-vocoded speech (HS < MS < LS). Second, if adults capitalize upon top-down lexical knowledge, both matching text primes and the lexical content of the target utterance would enhance the perceived speech clarity for noise-vocoded speech. Third, if spectral resolution and top-down processes interact as predicted above, we would see the greatest impact of top-down processing in conditions of higher spectral resolution. Our final hypothesis related to aging was that older adults would be able to utilize lexical information in conditions of more favorable signal quality, but its use would be relatively more restricted in conditions of poorer signal quality. We further explored if lexical information delivered visually (i.e., matching text prime) or auditorily (i.e., lexical content) would differentially impact speech clarity in older adults, depending on age-related declines in auditory sensitivity or neurocognitive functioning.

## Materials and Methods

### Participants

A total of 36 younger adults (17 female) and 38 older adults (26 female) were recruited for the current study. Younger participants were between the ages of 20–39 years and older participants were between the ages of 50–77 years, all with self-reported NH. All participants were recruited from the Prolific recruitment service (Prolific, 2021), an international research recruitment service. During testing, participants completed a short headphone screener to ensure the use of good-quality headphones during testing. Prior to analysis, six younger participants and eight older participants were excluded for failing a headphone screener. Thirty younger and older participants passed the headphone screener, with a score of ≥5 correct answers out of 6. The younger listener group (YNH) consisted of the remaining 30 younger participants (12 female), who were between the ages of 19 and 39 years (*M* = 29.8, *SD* = 5.8). The older listener group (ONH) consisted of the remaining 30 older participants (21 female), who were between the ages of 50 and 71 years (*M* = 57.3, *SD* = 5.8). All participants were native speakers of American English with no history of speech or language disorders. All participants provided electronic informed written consent prior to participation and received $7.50 for approximately 45 min of their time. Institutional Review Board (IRB) approval was obtained.

### Materials

Stimulus materials consisted of 144 sentences originating from the VAST sentence materials (Bell and Wilson, 2001), later recorded as part of the Multi-talker Corpus of Foreign-Accented English (MCFAE; Tamati et al., 2011). Sentences were produced by a female native speaker of American English from the Midland dialect region. At the time of the recording collection, the talker was 22 years old and reported no prior history of speech or hearing disorders.

Each VAST sentence contained three key words; all key words were either high or low lexical frequency and either high or low phonological neighborhood density. The total set of sentences contained 36 sentences with high lexical frequency and high neighborhood density key words (HH), 36 sentences with high lexical frequency and low neighborhood density key words (HL), 36 sentences with low lexical frequency and high neighborhood density key words (LH), and 36 sentences with low lexical frequency and low neighborhood density key words (LL). The sentence materials and key lexical properties are provided in the Supplementary Materials.

The three vocoder conditions were created by processing sentences through an 8-channel noise-band vocoder in Matlab with code maintained by the dB SPL lab at the University Medical Center Groningen (e.g., Gaudrain and Başkent, 2015). For all vocoding conditions, the original signal was filtered into 8 analysis bands between 150 and 7,000 Hz, using 12th order (72 dB/oct.), zero-phase Butterworth filters. The bands corresponded to evenly spaced regions of the cochlea using Greenwood’s frequency-to-place mapping function (Greenwood, 1990). The frequency cutoffs of individual bands were 150–301, 301–523, 523–852, 852–1,338, 1,338–2,056, 2,056–3,117, 3,117–4,684, and 4,684–7,000 Hz. The synthesis filters were 12th order filters (72 dB/octave) for the LS condition, 8th order filters (48 dB/octave) for the MS condition, and 4th order filters (24 dB/octave) for the HS vocoding condition, in order of decreasing spectral resolution. The synthesis filters had the same cutoff frequencies as the analysis filters. From each analysis band, the temporal envelope was extracted by half-wave rectification and low-pass filtering with a cutoff frequency of 300 Hz, using a zero-phase 4th order Butterworth filter (Gaudrain and Başkent, 2015). Noise carriers in each channel were modulated with the corresponding extracted envelope, which were then filtered by the synthesis filters. The modulated noise bands from all vocoder channels were added together to construct the stimuli. After processing, all stimuli were normalized to the same root mean square power.

### Procedure

Participants completed the experiment on the gorilla.sc platform using their own desktop or laptop devices and headphones. They were asked to sit in a quiet room and use good-quality headphones during the experiment. Prior to starting the speech clarity task, participants completed a language background questionnaire, the headphone screener, and a familiarity block. The online headphone screener consisted of a three-alternative forced-choice task in which participants listened to three white noise sounds (all 1,000 ms) – one of which contains a faint tone – at comfortable level and responded as to which sound contained the tone (Milne et al., 2020). The headphone test was designed based on Huggins Pitch, a dichotic pitch percept that should be detectable only when using headphones. For each trial, two intervals contained diotically-presented white noise. The third interval contained the target Huggins Pitch stimulus. The percept of pitch was generated by presenting a white noise stimulus to one ear and the same white noise with a phase shift of 180 degrees over a narrow frequency band around the center frequency of 600 Hz to the other ear. The result of this manipulation is the perception of a tone with the pitch of the center frequency of the phase-shifted band (i.e., 600 Hz) in noise. For the purpose of the current study, participants who scored <5 correct answers out of 6 were considered to have failed the headphone screener and were excluded from the analysis. Although not designed to screen for other aspects of the listener’s environment, participants completing the experiment in a noisy environment and/or participants with hearing impairment may also fail this screener (e.g., Santurette and Dau, 2007). During the familiarity block, participants were able to gain familiarity with noise-vocoded speech and the ratings scale. Three vocoded sentences were presented to provide references for the ratings scale of 1 (“very unclear”) to 7 (“completely clear”). A LH sentence in the HS vocoder condition was used as a reference for low clarity, a HL sentence in the LS condition was used as a reference for high clarity, and a HH sentence in the MS condition was used as a reference for the middle range.

On each trial of the main speech clarity task, listeners were presented with a single sentence and were asked to rate the clarity of each sentence on a scale from 1 (“very unclear”) to 7 (“completely clear”). The sentence was always preceded by a 500 ms fixation cross on the computer screen and a matching or non-matching text prime. The text prime appeared on the screen for 2.5 s in order to allow the participant enough time to read the text. The matching prime consisted of the word-by-word orthographic transcription of the target sentence. The non-matching prime was created by randomly reorganizing the letters of the original prime into nonsense words; non-matching primes were controlled to ensure that no real words resulted from the randomization. After reading the text prime, listeners were presented with the target sentence and responded by clicking one of seven numerical response options. Forty-eight sentences (12 of each HH, HL, LH, and LL) were presented in each of the LS, MS, and HS vocoder conditions. Half of the sentences (6 of each sentence type in each vocoder condition) were preceded by a matching text prime, while the other half of the sentences were preceded by a non-matching text prime. Rating responses were recorded and coded by sentence type, vocoder, and text prime condition.

## Results

A mixed ANOVA on speech clarity ratings was carried out with vocoder condition (LS, MS, and HS), priming (matching and non-matching), and sentence type (HL, HH, LL, and LH) as within-subject factors and listener group (YNH and ONH) as the between-subject factor. An alpha of 0.05 was used. *Post hoc* Tukey tests were used to explore the significant main effects and interactions. Significant main effects of vocoder [*F*(2,58) = 414.09, *p* < 0.001, $\eta_{\text{p}}^{2}$ = 0.88], priming [*F*(1,58) = 150.61, *p* < 0.001, $\eta_{\text{p}}^{2}$ = 0.72], and sentence type [*F*(3,174) = 84.61, *p* < 0.001, $\eta_{\text{p}}^{2}$ = 0.59] emerged, indicating that both the bottom-up factor of vocoder (i.e., spectral resolution) and the top-down factors of priming and sentence type impacted the perceived clarity of noise-vocoded speech. For vocoder condition, clarity ratings were significantly higher (i.e., perceived as clearer) in the LS (*M* = 4.91, *SD* = 0.76) and MS conditions (*M* = 5.04, *SD* = 0.77) compared to the HS condition (*M* = 3.43, *SD* = 0.83) (all *p* < 0.001). However, the LS and MS conditions were not significantly different from one another. For priming condition, clarity ratings were also significantly higher with matching (*M* = 4.96, *SD* = 0.83) than non-matching text primes (*M* = 3.96, *SD* = 0.77) (*p* < 0.001). For sentence type condition, clarity ratings were significantly higher in the HL condition (*M* = 4.68, *SD* = 0.75) compared to the LH conditions (*M* = 4.09, *SD* = 0.79) (*p* < 0.001). Clarity ratings were also significantly higher in the HH (*M* = 4.63, *SD* = 0.77) than the LH condition (*p* < 0.001), and in the LL (*M* = 4.45, *SD* = 0.74) compared to the LH condition (*p* < 0.001). No other comparisons reached significance. These results confirm the effects of the spectral resolution, matching text primes, and the lexical properties of frequency and neighborhood density on clarity ratings for noise-vocoded speech. The main effect of group was not significant (YNH: *M* = 4.43, *SD* = 0.68; ONH: *M* = 4.49, *SD* = 0.81), suggesting a lack of overall differences in clarity ratings between younger and older adults.

**FIGURE 1:**
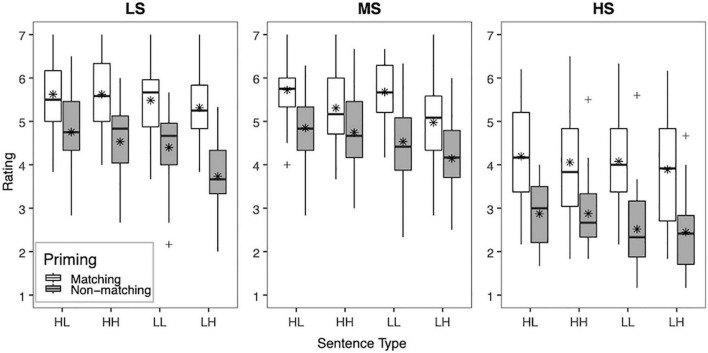
Box plot showing the mean clarity ratings for NH younger adults (YNH). Clarity ratings (1 “very unclear” to 7 “completely clear”) are plotted across all three vocoder conditions (LS, MS, and HS), priming condition (matching and non-matching), and sentence type (HL, HH, LL, and LH). The boxes extend from the lower to the upper quartile (the interquartile range, IQ), the solid midline indicates the median, and the star indicates the mean. The whiskers indicate the highest and lowest values no greater than 1.5 times the IQ, and the plus signs indicate outliers, which are defined as data points larger than 1.5 times the IQ.

**FIGURE 2:**
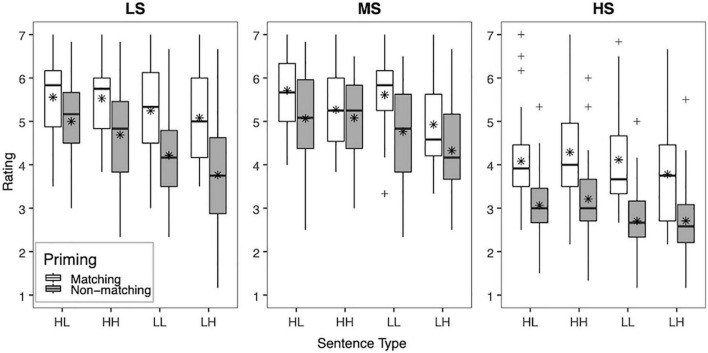
Box plot showing the mean clarity ratings for NH older adults (ONH). Clarity ratings (1 “very unclear” to 7 “completely clear”) are plotted across all three vocoder conditions (LS, MS, and HS), priming condition (matching and non-matching), and sentence type (HL, HH, LL, and LH). The boxes extend from the lower to the upper quartile (the interquartile range, IQ), the solid midline indicates the median, and the star indicates the mean. The whiskers indicate the highest and lowest values no greater than 1.5 times the IQ, and the plus signs indicate outliers, which are defined as data points larger than 1.5 times the IQ.

To facilitate interpretation of the interactions among the factors, the mean clarity ratings across vocoder conditions (LS, MS, and HS) in each priming condition (matching, non-matching) and sentence type (HL, HH, LL, and LH) are shown for YNH listeners in Figure 1 and ONH listeners in Figure 2. Significant two-way interactions of vocoder × priming [*F*(2,116) = 9.17, *p* < 0.001, $\eta_{\text{p}}^{2}$ = 0.39] and vocoder × sentence type [*F*(6,348) = 7.84, *p* < 0.001, $\eta_{\text{p}}^{2}$ = 0.12] were uncovered, suggesting that the strength of effects of the top-down factors of priming and sentence type depended on the bottom-up factor of vocoder (i.e., spectral resolution). For priming condition, clarity ratings were higher with matching than non-matching primes for all vocoder conditions (all *p* < 0.001). The effect of sentence type differed by vocoder condition. For the HS condition (i.e., low spectral resolution), no significant differences among sentence types emerged. For the MS condition (i.e., medium spectral resolution), ratings were higher for the HL, HH, and LL conditions than the LH condition (all *p*≤0.002), but no other comparison reached significance. For the LS condition (i.e., highest spectral resolution), ratings were higher for the HL and HH conditions than the LH condition (all *p* < 0.001), but no other comparisons among sentence types reached significance.

A significant two-way priming × sentence type interaction [*F*(3,174) = 11.03, *p* < 0.001, $\eta_{\text{p}}^{2}$ = 0.16] also emerged, suggesting that the effect of sentence type depended on the availability of visual text priming. Overall, for the matching priming condition, no comparison reached significance. For the non-matching priming condition, clarity ratings were significantly higher for the HL and HH conditions than the LH condition (all *p* < 0.001), and for the HL condition than the LL condition (*p* = 0.036). However, the three-way interaction of vocoder × priming × sentence type was also significant [*F*(6,348) = 7.12, *p* < 0.001, $\eta_{\text{p}}^{2}$ = 0.11], suggesting that the effect of lexical content depended both on the priming and vocoder condition. For the HS condition (i.e., low spectral resolution), no significant differences among sentence types emerged for either the matching or non-matching priming condition. For the MS condition (i.e., medium spectral resolution), clarity ratings for the LL condition were higher than for the LH condition for the matching priming condition (*p* = 0.035). For the non-matching priming condition, clarity ratings for the HL condition were higher than for the LH condition (*p* = 0.023). For the LS condition (i.e., highest spectral resolution), no significant differences among sentence types emerged for the matching priming condition. For the non-matching priming condition, clarity ratings for the HL and HH conditions were higher than for the LH condition (all *p* < 0.001). To summarize, these comparisons suggest that sentence type had little to no effect on clarity ratings for the HS vocoder condition. However, sentence type had an effect on clarity ratings for both matching and non-matching priming conditions for the MS condition, and for non-matching primes for the LS condition. Additionally, the most consistent difference among sentence types was observed between HL and LH conditions.

## Discussion

The current study investigated top-down lexical effects on the perceived clarity of noise-vocoded speech, and interactions with bottom-up signal quality, in NH younger and older adults. More specifically, the current study examined form-based priming, by introducing matching or non-matching text primes presented prior to the target utterance, as well as the lexical content of the target utterance, by varying the lexical frequency and neighborhood density of key words. Given that the current study was conducted online, we also sought to determine if speech clarity ratings from the online task were consistent with previous studies using an in-person experimental procedure (e.g., Signoret et al., 2018; Signoret and Rudner, 2019).

Examining the effects of bottom-up signal quality, we hypothesized that decreasing spectral resolution would result in lower perceived clarity of noise-vocoded speech. Consistent with our hypothesis, a main effect of vocoder condition demonstrated that increased spectral resolution enhanced the perceived clarity of noise-vocoded speech in both NH younger and older adults. These findings are broadly consistent with results from previous in-person studies (Signoret et al., 2018; Signoret and Rudner, 2019), in which spectral resolution was varied by manipulation of the number of vocoder channels. In the current study, an 8-channel vocoder was used to simulate the same number of electrode contact points in each vocoder condition, but spectral resolution was varied by manipulating the sharpness of the bandpass filter slopes to simulate low, medium, and high spread of excitation in the cochlea. Decreased spectral resolution via simulation of increased channel interaction has been found to result in less accurate speech recognition (Fu and Nogaki, 2005; Bingabr et al., 2008; Wang et al., 2012; Oxenham and Kreft, 2014; Winn et al., 2016; Mehta et al., 2020), less accurate pitch perception (Crew et al., 2012; Mehta and Oxenham, 2017), increased listening effort (Winn et al., 2016), and limitations in the perception of non-linguistic aspects of speech, such as voice cue perception (Gaudrain and Başkent, 2015; Koelewijn et al., 2021). In the current study, while there was a main effect of vocoder condition, only differences between the condition with the worst spectral resolution (HS; 4th order, 24 dB/octave) and the conditions with increasingly more favorable spectral resolutions (MS and LS; 8th order, 48 dB/octave and 12th order, 72 dB/octave, respectively) emerged. Similarly, Gaudrain and Başkent (2015) found that improving spectral resolution by increasing the filter order from 4 (24 dB/octave) to 8 (48 dB/octave) in a 12-channel noise-vocoder improved perception of vocal tract length cues, but further increasing to 12 (72 dB/octave) did not improve perception. In the current study, sharpening the filter slopes beyond 8 (48 dB/octave) also did not drastically enhance the perceived clarity of noise-vocoded speech when using eight channels. Although consistent with previous in-person studies, the extent to which clarity ratings were impacted by the online administration of the task is unclear. With the online study, the testing environment and equipment were not controlled. Additionally, participants’ hearing thresholds were not evaluated. Although participants completed a headphone screener, the screener was not designed to specifically evaluate these factors. It is possible that better controlling for these factors in an in-person setting would result in clarity ratings that are more sensitive to subtle differences in spectral resolution. Here, findings suggest that younger and older adults perceived 8-channel noise-vocoded speech as clearer with improved spectral resolution introduced by increasing the sharpness of the filter slopes from the HS to MS and LS vocoder conditions. More controlled studies should be carried out in the future to further investigate the effects of bottom-up signal quality on the perceived clarity of noise-vocoded speech.

To investigate how top-down lexical knowledge affects the perceived clarity of noise-vocoded speech, we explored the effects of form-based prediction and varying lexical content (i.e., lexical frequency and neighborhood density) on the perceived clarity of noise-vocoded speech. Consistent with previous in-person studies (Wild et al., 2012; Signoret et al., 2018; Signoret and Rudner, 2019), results demonstrate a clarity-enhancing effect of form-based prediction. In their studies, Signoret et al. (2018) and Signoret and Rudner (2019) similarly demonstrated that matching text presented visually prior to the target utterance enhances the clarity of noise-vocoded speech in NH younger and hearing-impaired older listeners. Here, degraded speech was perceived as being clearer when a matching text prime had been presented prior to its auditory presentation, compared to when degraded speech was preceded by a random, meaningless assortment of letters. Moreover, the benefit from matching text primes was observed in all vocoder conditions, for both younger and older adults. Matching text provides identical lexical and phonological content of the utterance, and reliably facilitates the activation of the exact lexical items in the target utterance. As such, presenting text that partially or exactly matches the lexical and phonological content of the target utterances enhances the recognition and perceived clarity of degraded speech by allowing the listener to generate expectations about the upcoming target utterance (Wild et al., 2012; Sohoglu et al., 2014; Signoret et al., 2018; Signoret and Rudner, 2019).

To expand upon previous findings and further investigate top-down lexical effects, we also examined how the lexical content of the target utterance impacts the perceived clarity of noise-vocoded speech. We tested the hypothesis that sentences containing high frequency words with few phonological neighbors (low neighborhood density) would be perceived as clearer than sentences containing low frequency words with many phonological neighbors (high neighborhood density). Consistent with our hypothesis, we found a significant main effect of sentence type in the overall analyses across vocoder conditions, as well as significant effects of sentence type in each vocoder condition when exploring the interactions. Although sentence types that emerged as significantly different from one another varied by vocoder and priming conditions, sentences containing lexically easy words (high lexical frequency and low neighborhood density) were consistently perceived as clearer than sentences containing lexically hard words (low lexical frequency and high neighborhood density) by both younger and older listener groups. For example, HL, HH, and LL sentences were overall rated as clearer than LH sentences. Given that the most consistent difference emerged between the easy (i.e., HL) and hard (i.e., LH) sentences, these findings suggest a role for both lexical frequency and neighborhood density in the perceived clarity of noise-vocoded speech for younger and older adults.

Additionally, the lexical content of sentence key words (i.e., lexical frequency and neighborhood density) contributed to clarity ratings both when supportive visual information was available (matching text primes) and when listeners had to rely on auditory information alone (non-matching text primes). Thus, lexical content was utilized with the support of both combined visual and auditory information as well as auditory information alone in younger and older adults. The overall effects of lexical frequency and neighborhood density are consistent with existing accounts of spoken word recognition that emphasize the integration of top-down lexical knowledge with bottom-up acoustic-phonetic details during spoken word recognition, such as the Neighborhood Activation Model (NAM; Luce et al., 1990; Luce and Pisoni, 1998). Previous findings consistent with these accounts have demonstrated that lexically easy words are recognized or discriminated more accurately and faster than hard words under noise-vocoding (e.g., Tamati et al., 2020b) as well as in hearing-impaired listeners with or without cochlear implants (e.g., Dirks et al., 2001; Takayanagi et al., 2002; Tamati et al., 2021). Further, some evidence suggests that lexical content differentially facilitates reaction time in shadowing tasks when participants are presented with partially or exactly matching auditory primes (Dufour and Peereman, 2004), since easy target words are quickly activated from the prime prior to hearing the target and remain activated due to phonological overlap between the prime and target. Our findings extend upon these previous studies by demonstrating that these lexical properties impact the perceived clarity of noise-vocoded speech in younger and older adults.

Another goal of the study was to investigate how top-down lexical knowledge interacts with bottom-up signal quality. We hypothesized that an interaction between bottom-up and top-down processing would result in a decreased contribution of lexical knowledge on perceived clarity of sentences with relatively poor signal quality (HS), relative to conditions with relatively better quality (MS and LS). Indeed, the contribution of lexical knowledge described above appears to vary based on the degree of degradation of the noise-vocoded speech. However, in contrast with our initial hypothesis, the benefit from matching text primes was observed in all vocoder conditions, for both younger and older adults. However, a greater relative effect of priming appeared to emerge for the HS condition, which provided the most degraded spectral resolution, as can be seen in Figures 1, 2. Similarly, Signoret et al. (2018) found that form-based prediction had a stronger effect at lower degrees of signal quality (3-channel noise vocoder). Although not designed to test these accounts, our results are consistent with accounts of degraded speech recognition, such as the Ease of Language Understanding Model (ELU; Rönnberg et al., 2013), which emphasizes the role of top-down processing when bottom-up processing is insufficient. It is worth pointing out that form-based predictions about the upcoming target utterance were generated based on visual text information, which was not degraded either visually or auditorily. As such, the matching text prime provided a reliable source of lexical information that enhanced the clarity of the noise-vocoded speech, regardless of the degree of degradation of the target utterance. Similarly, other sources of linguistic information that remain unaltered despite degradation in signal quality, such as visual contextual cues relating to the setting of a conversation (e.g., formal or informal; Brouwer et al., 2012) or text information from subtitles (Mitterer and McQueen, 2009), may be relied upon to enhance speech clarity and facilitate spoken word recognition in real-world, adverse conditions.

Broadly consistent with our initial hypothesis, the lexical content of the utterance appeared to contribute less to the perceived clarity of noise-vocoded speech in the HS condition, where speech was more degraded, and relatively more in the MS and LS conditions, where speech was less degraded. While sentence type (i.e., HL, HH, LL, and LH) was significant overall in the HS condition, differences did not emerge among individual sentence types. Insights into how bottom-up signal quality may have influenced the relative reliance on lexical content can be obtained by specifically examining the contribution of sentence type (i.e., lexical content) with and without matching text primes. In the LS condition (higher spectral resolution), sentence type only contributed to perceived sentence clarity without matching text primes; easy words were perceived as clearer than hard words only when participants had to rely upon the auditory signal alone for both younger and older adults. In the MS condition (middle spectral resolution), sentence type influenced perceived speech clarity both with and without matching primes. Overall, these findings demonstrate that sentence type had less influence on perceived clarity when spectral resolution was poor and potentially when combined conditions were among the most favorable (highest spectral resolution combined with matching text primes). Thus, top-down use of lexical content may be most relevant in conditions of moderate degradation.

These findings are largely consistent with previous research showing that top-down compensation may become less effective when the degree of degradation of the speech signal is more extreme (Samuel, 1981; Król and El-Deredy, 2011; Bhargava et al., 2014; Sohoglu et al., 2014), and reliance on top-down processing may decrease (Mattys et al., 2009; Clopper, 2012). Similarly, in the HS condition in the current study, the degraded speech signal likely did not provide sufficient acoustic-phonetic detail to support the robust use of top-down lexical knowledge (Aydelott and Bates, 2004; Mattys et al., 2005, 2009; Clopper, 2012). In other words, in the current study, listeners did not rely on the lexical content to the same extent in conditions of poor signal quality compared to conditions of more favorable signal quality, when lexical information was delivered solely by the degraded target utterance. These findings are consistent with previous studies showing that individual CI users with poorer bottom-up signal quality may less effectively employ top-down compensatory mechanisms to process the degraded speech delivered by the CI (Bhargava et al., 2014; Tamati et al., 2020a; Moberly et al., 2021). In contrast with matching text primes, lexical content delivered by a degraded speech signal, as well as other forms of top-down linguistic information such as semantic context, may be susceptible to bottom-up signal quality and may not be engaged to facilitate speech understanding as effectively in real-world, adverse conditions. Taken together, our findings suggest that the HS condition provided such a poor signal that only matching text primes could largely be relied upon to enhance perceived speech clarity; in contrast, in the LS condition, the lexical content of auditorily presented target utterance could be relied upon to a greater extent. Thus, top-down lexical knowledge was employed with the support of both combined visual and auditory information as well as auditory information alone in younger and older adults, and further interacts with bottom-up signal quality to impact the perceived clarity of noise-vocoded speech. However, these findings should be interpreted with caution. As mentioned above, the current study did not control for several factors, including the testing environment (e.g., noise or distractors) or the audiometric thresholds of the listeners, that could have impacted the quality of the signal conveyed to individual listeners and the relative reliance on top-down mechanisms. Future studies that better control for these factors are needed to shed more light on the interaction of bottom-up and top-down processing.

The final hypothesis tested in the current study related to the effects of aging on top-down processing. Overall, we predicted that older adults would effectively utilize top-down lexical knowledge to the same extent, if not more, than younger adults, at least in conditions of more favorable signal quality. However, we further predicted that if the use of top-down lexical knowledge is restricted by poorer auditory and/or cognitive functioning, then older adults would not demonstrate strong clarity-enhancing effects of matching text primes and lexical content in conditions of poorer signal quality. The general finding here did not support that hypothesis. Instead, both younger and older listener groups showed similar effects of matching text primes and lexical content on speech clarity ratings consistently across degrees of signal degradation. Regarding form-based prediction, the clarity-enhancing benefit observed from matching text primes was similar in the younger and older listener groups. Our findings provide additional evidence that degraded speech is perceived as clearer when the listener is provided with text that matches the target utterance prior to its auditory presentation (e.g., Sohoglu et al., 2012, 2014; Wild et al., 2012; Wu et al., 2012; Getzmann et al., 2014; Signoret et al., 2018; Signoret and Rudner, 2019). Additionally, Signoret et al. (2018) found the ability to use form-based prediction as well as semantic context was related to working memory capacity, suggesting a role for cognitive abilities in top-down compensation and a potential means by which aging could affect the perceived clarity of noise-vocoded speech. Although outside the scope of the current study, one potential explanation for the similarity of form-based prediction between the younger and older listeners could be that working memory capacity did not differ between our groups, or working memory capacity was not implicated within the speech clarity rating paradigm used currently. Moreover, while conducting the study using an online experimental protocol may have enabled recruitment from a larger participant pool, both the younger and older adults would have been comfortable with technology and online research. Thus, findings may not generalize broadly to other populations Yet, importantly, the current study expands on that literature to show that these priming effects do not appear to deteriorate significantly with aging.

Regarding lexical content, the results of the current study also showed that the younger and older adults appeared to demonstrate similar combined effects of lexical frequency and neighborhood density. These findings are in line with work by Taler et al. (2010), who examined the effects of lexical competition on word-in-sentence recognition. There, both groups of older and younger adults recognized words in sentences more accurately and quickly for sentences containing high frequency words (vs. low frequency words) as well as for sentences containing words with low neighborhood density (vs. high neighborhood density). In contrast, our findings differ somewhat from studies suggesting that older listeners display more difficulty in resolving lexical competition during speech recognition (Sommers, 1996; Sommers and Danielson, 1999; Helfner and Jesse, 2015). In addition, some previous studies have suggested that older adults rely more heavily on lexical frequency than younger adults in both auditory speech perception (Revill and Spieler, 2012) and visual word processing (Spieler and Balota, 2000; Balota et al., 2004). Notably, several of the studies examining neighborhood density effects have at least partially attributed these age-related differences to poorer inhibitory control in older listeners (e.g., Sommers and Danielson, 1999). For example, in the Taler et al. (2010) study, difference scores between accuracy for words with high and low neighborhood density at a lower SNR (−3 dB SNR) were negatively related to inhibitory control across all listeners. Additionally, changes in lexical processing across the lifespan may also be attributable to increases in vocabulary size with aging (e.g., Salthouse, 2004; McAuliffe et al., 2013; Ramscar et al., 2014; Carroll et al., 2016). Although we did not assess inhibitory control or vocabulary size in this study, a potential explanation for the similarity of neighborhood density effects between the younger and older listeners could be that inhibitory control or vocabulary size did not differ substantially between groups. Relatedly, the older participants in the current study were slightly of a younger age, with a mean age of 57.3 years and a range of 50–71 years, compared to the studies that have observed differences in lexical processing between younger and older listeners. Previous studies have included groups of older adults with mean ages of around 65–75 years. Finally, another possibility is that the current outcome measure – online perceptual ratings of speech clarity – was not sensitive to differences in inhibitory control or vocabulary size between the younger and older listening adults. In Taler et al. (2010), for example, the dependent measures were response times and accuracy in sentence recognition tasks, both of which may involve different levels of lexical-phonological processing than would be expected in the speech clarity rating paradigm used presently. Future studies examining the effects of aging on top-down compensation should consider using alternative measures, and including a wider age range of older participants.

In addition to examining the overall impact of aging on top-down compensation, we also considered two additional alternative hypotheses: that if poorer neurocognitive functioning contributes to an aging-related deficiency in top-down processing, then older adults would be limited in their use of lexical information, regardless of the modality of the source (i.e., matching text primes presented visually and lexical content presented auditorily). The alternative hypothesis was that if poorer auditory sensitivity contributes to an aging-related deficiency, then older adults should not show strong effects of lexical content on the clarity of noise-vocoded speech specifically when relying exclusively on auditory information (i.e., with a non-matching prime). In other words, there may be differences between age groups in which top-down mechanisms would enhance speech clarity. Previous studies from Signoret et al. (2018) and Signoret and Rudner (2019) identified potential differences in the interaction of top-down and bottom-up processes in younger and older, hearing-impaired adults, who seemed to exhibit less top-down compensation with more severe degrees of degradation, at which the younger adults had benefited. In contrast, our results suggested overall very similar effects of matching text primes and lexical content for the younger and older groups.

More generally, findings from this study provide additional evidence that older listeners can effectively enhance the processing of a novel form of degraded speech by making use of their crystallized intelligence (here and lexical knowledge), which has been found to be maintained into older age (Salthouse, 1993; Wingfield et al., 1994; Ryan et al., 2000; Park et al., 2002). Our findings are therefore consistent with previous studies showing that older adults can capitalize on crystallized intelligence in adverse listening conditions to the same extent as younger listeners (e.g., Balota and Duchek, 1991; Wingfield et al., 1994; Pichora-Fuller et al., 1995; Valencia-Laver and Light, 2000; Daneman et al., 2006; Sheldon et al., 2008). However, more research should be carried out to better understand top-down mechanisms and how their effective use may depend on bottom-up signal quality and age, particularly for older CI users who must deal with a degraded speech signal as part of their normal, daily communication.

### Clinical Implications for Adult Cochlear Implant Users

The findings from the current study may have implications for understanding and addressing the vast individual differences in speech recognition outcomes observed among adult CI users (Lazard et al., 2012; Lenarz et al., 2012; Blamey et al., 2013). First, the preservation of top-down processing with advancing age is highly significant because it suggests that older listeners compensate for degraded listening conditions using their long-term linguistic knowledge. Therefore, targeting the use of linguistic context in understanding speech in rehabilitative training may be effective in helping adult CI users across the lifespan achieve real-world communication success. Second, our findings suggest that the effective use of some top-down compensatory mechanisms may crucially depend on bottom-up signal quality. This finding is clinically relevant since it could suggest that some top-down compensatory strategies across individual CI users may crucially depend on the quality of the bottom-up input. More specifically, similar to findings from Tamati et al. (2020a) and Moberly et al. (2021), individual CI users with poor bottom-up auditory input may not be able to take advantage of some top-down resources to effectively compensate for the degraded speech delivered by their CIs.

However, the relevance of the findings to CI users should be interpreted with caution. The current study used acoustic noise-vocoder CI simulations to simulate degraded speech that captures functional performance of adult CI users. Acoustic simulations capture the basic signal processing steps of CIs (Loizou, 1998) and, for some spoken word recognition tasks, the performance ranges of actual CI users (e.g., Friesen et al., 2001). However, there are many factors that additionally affect speech perception in CI users (Başkent et al., 2016b), including the severity and duration of deafness prior to implantation, and duration of CI use (e.g., Blamey et al., 2013). Increased severity and longer durations of deafness prior to implantation have been linked to weak phonological processing and poorer speech recognition outcomes in adult CI users (Lyxell et al., 1998; Lazard et al., 2010; Lazard and Giraud, 2017; Tamati et al., 2021). Weakened phonological processing may impact the structure and organization of the mental lexicon, thereby altering how or the extent to which listeners utilize lexical knowledge, issues we would not expect to face when testing NH younger and older adults. Additionally, CI users appear to benefit from experience using their devices to more effectively use top-down compensatory mechanisms (e.g., Winn et al., 2012; Fuller et al., 2014; Bhargava et al., 2016). In contrast, the NH listeners in the current study had minimal experience with noise-vocoded speech prior to testing. NH adults typically adapt quickly to noise-vocoded speech and reach a stable level of recognition accuracy with a small number of sentences (Davis et al., 2005; Hervais-Adelman et al., 2008; Huyck et al., 2017), particularly when presented with matching text primes (Davis et al., 2005). Nevertheless, the novelty of this form of degradation may have altered the reliance on top-down lexical knowledge. Thus, additional studies, possibly involving more diverse younger and older listeners with or without CIs, along with measures of demographic and cognitive-linguistic abilities, are needed to better understand the roles of top-down and bottom-up processing on the perception of degraded speech.

## Conclusion

The current study examined how top-down cognitive-linguistic and bottom-up sensory factors affect the perceived clarity of speech in younger and older adults using an online speech clarity task. Findings demonstrate that both younger and older adults were able to effectively use lexical knowledge to enhance the clarity of noise-vocoded speech. In particular, listeners perceived the speech as clearer when preceded by an exact matching text prime and when the target utterance contained lexically easy words (i.e., high lexical frequency and low neighborhood density) compared to hard words (i.e., low lexical frequency, high neighborhood density). However, the effective use of top-down lexical knowledge appeared to depend on the bottom-signal quality. While matching text primes provided a relatively greater enhancement of more degraded speech, lexical content had a greater impact with more moderately degraded speech. Importantly, these findings also show that older adults make use of lexical knowledge to a similar degree as the younger listeners. Taken together, these findings emphasize the interactive nature of bottom-up and top-down processes in the perception of degraded speech. Further, findings suggest that lexical knowledge could be effectively used to enhance speech understanding in adult CI users across the lifespan, but some CI users may be hindered by a relatively poor signal.

## Data Availability Statement

The raw data supporting the conclusions of this article will be made available by the authors, without undue reservation, to any qualified researcher.

## Ethics Statement

The studies involving human participants were reviewed and approved by Institutional Review Board at Ohio State University. The patients/participants provided their written informed consent to participate in this study.

## Author Contributions

TT designed and conducted the study. TT, VS, and AM contributed to data analysis and interpretation. All authors contributed to the writing of the manuscript and approved of the final version of the manuscript for submission.

## Conflict of Interest

AM and TT have received grant funding support from Cochlear Americas for unrelated investigator-initiated research studies. AM has served as a paid consultant for Cochlear Americas and Advanced Bionics and is CMO and on Board of Directors for Otologic Technologies. The remaining authors declare that the research was conducted in the absence of any commercial or financial relationships that could be construed as a potential conflict of interest.

## Publisher’s Note

All claims expressed in this article are solely those of the authors and do not necessarily represent those of their affiliated organizations, or those of the publisher, the editors and the reviewers. Any product that may be evaluated in this article, or claim that may be made by its manufacturer, is not guaranteed or endorsed by the publisher.
