# Supplementary material for: Lexical Effects on the Perceived Clarity of Noise-Vocoded Speech in Younger and Older Listeners
Source: Front Psychol. 2022 Apr 1;13:837644. doi: 10.3389/fpsyg.2022.837644 (PMC9010567; doi:10.3389/fpsyg.2022.837644)
Supplement: Supplementary file 1 [file Table_1.DOCX]

Supplementary Material

**Sentence List**

VAST sentences used in the current study. Keywords are underlined and bolded. Within each sentence, all keywords either have a high (H) or low (L) lexical frequency and a high or low number of neighbors (neighborhood density).

| Lexical Frequency | Neighborhood Density | Sentence |
| --- | --- | --- |
| H | L | The **point** of the **knife** is too **sharp**. |
| H | L | The **model** wore a **plain** black **dress**. |
| H | L | The **mouse** **fled** when it saw the light **flash**. |
| H | L | The **barrel** **burst** when it was too **full**. |
| H | L | The **worst** **stuff** got the lowest **price**. |
| H | L | It is a **risk** to **drive** through the **field**. |
| H | L | **Warn** the new **cook** that the eggs may **burst**. |
| H | L | The **breeze** helped to **clear** the **fog**. |
| H | L | He **flung** a **bottle** at the **crowd**. |
| H | L | **Claim** your **clothes** when I'm done with the **wash**. |
| H | L | **Grip** the **handle** as you lift the **drill**. |
| H | L | You **could** use the **switch** near the **lamp**. |
| H | L | They **plan** to drive a **truck** on their **tour**. |
| H | L | The **damp** soil made his **foot** look **brown**. |
| H | L | The **couple** gathered **nuts** in the **woods**. |
| H | L | His **bunk** was **flat** and made of **wood**. |
| H | L | I **swear** if you eat **toast** you'll stay **slim**. |
| H | L | **Move** over and **join** us on the **couch**. |
| H | H | The brass **band** went **home** to **rest**. |
| H | H | We **tried** to **sink** the British **fleet**. |
| H | H | Her **coat** got **wet** when she spilled her **wine**. |
| H | H | The first **half** of the **test** was **hard**. |
| H | H | He should **learn** not to **spit** and **curse**. |
| H | H | **Pull** a small **board** off of the **shed**. |
| H | H | She would not **dare** to **leave** the sick **man**. |
| H | H | The **map** is on the **deck** of the **ship**. |
| H | H | You **can't** **park** where they load **freight**. |
| H | H | The **tall** man **rose** when we **met**. |
| H | H | The **moon** in the sky **shone** on the **reef**. |
| H | H | He was **hit** on the **back** with a **bat**. |
| H | H | **Raise** the oven **rack** when you **bake**. |
| H | H | **Part** of the **stake** has been **bent**. |
| H | H | She **got** in **bed** when she hurt her **head**. |
| H | H | **Keep** the **soup** warm on the **fire**. |
| H | H | You must try to **learn** to **stand** **still**. |
| H | H | That **type** of **hawk** lives in the **park**. |
| L | L | The **lump** of **cheese** has turned **sour**. |
| L | L | He **knelt** to **greet** the king in the **castle**. |
| L | L | The bird will **swoop** down to **fetch** the **crumb**. |
| L | L | The **stag** has been **bitten** by the **hound**. |
| L | L | Use **slang** to scare **away** the **flirt**. |
| L | L | The **wind** will **rustle** the flap on the **bib**. |
| L | L | She could not **breathe** in her **snug** new **girdle**. |
| L | L | Use a **gavel** to **smash** the **pebble**. |
| L | L | The three-**legged** dog would **yelp** as it **swam**. |
| L | L | The **thieves** were **hanged** for stealing **flour**. |
| L | L | A **throng** of people **leapt** on the **shuttle**. |
| L | L | The **clown** will **juggle** to make the kids **giggle**. |
| L | L | **Dab** off the ink when you **dunk** the **quill**. |
| L | L | **Play** a psalm on your **flute** near the **blaze**. |
| L | L | The **thieves** were **hanged** for stealing the **jewel**. |
| L | L | **Grease** will make the **raft** feel **slick**. |
| L | L | He **shaves** with **knives** and soap **suds**. |
| L | L | The **clown** will **baffle** the kids till they **giggle**. |
| L | H | **Water** came from the **leak** in the **dike**. |
| L | H | The **foul**-smelling **pig** likes to **wade**. |
| L | H | The **lamb** likes to **lick** the salt **loaf**. |
| L | H | The **rock** in the **sling** can wreck the **kite**. |
| L | H | The **dent** in his new **bike** made him **yell**. |
| L | H | **Coil** the **hose** neatly near your **bike**. |
| L | H | The **canned** fruit made a **stain** on his **cuff**. |
| L | H | He likes to **poke** his **pet** **rat**. |
| L | H | Mash the **peach** in a **bowl** for the **pup**. |
| L | H | The **crowd** will jeer as they **mock** the **hag**. |
| L | H | **Chop** a **rib** from one side of the **pig**. |
| L | H | **Nip** the **bud** if you see a **bug**. |
| L | H | Hang the **cage** on a **hook** in the **yacht**. |
| L | H | He will **sob** if you **wreck** his new **bike**. |
| L | H | **Kneel** as you **reel** in the **bait**. |
| L | H | She will **yell** if you **tug** on her **braid**. |
| L | H | The **dove** likes to **perch** on the top **rung**. |
| L | H | The **foal** likes to **lick** dried **sap**. |
| H | L | The **wives** were **tired** and had many **needs**. |
| H | L | The **yarn** in the **box** was **soft**. |
| H | L | The **flock** will **nest** in the wild **brush**. |
| H | L | The **small** **lights** were green and **purple**. |
| H | L | The **troop** can rest in the **warm** **lodge**. |
| H | L | The **guest** had to **dash** home for his **check**. |
| H | L | They put a **loud** **pump** near the **drain**. |
| H | L | The **curtain** **swung** open for the **dance**. |
| H | L | She tried to **steal** the **mound** of **silk**. |
| H | L | **Treat** the **group** to something **warm**. |
| H | L | He **stole** each **check** issued to the **gang**. |
| H | L | The **rebel** has a **large** horse to **mount**. |
| H | L | The **track** for the **short** race is the **worst**. |
| H | L | **Dash** to the **bank** ahead of the **mob**. |
| H | L | Try to **choose** a **plain**-looking **table**. |
| H | L | She soon **learned** that her **cousin** was a **burden**. |
| H | L | The **trace** of **green** ink was **bright**. |
| H | L | **Spare** the **soil** you need the **most**. |
| H | H | I **miss** all the **fun** that comes with **fame**. |
| H | H | He **will** not **pick** a wooden **cane**. |
| H | H | I went with the **team** to **find** a **tent**. |
| H | H | The **bus** headed **west** at **dawn**. |
| H | H | He **sank** the clay **cup** in the **tub**. |
| H | H | **Get** all the **soap** off your **face**. |
| H | H | The **mold** on the **book** made it **stick**. |
| H | H | She has **gone** to **lend** him a **jar**. |
| H | H | He **had** to **ride** with the **pack**. |
| H | H | Please **knock** when you **reach** the **tent**. |
| H | H | A **thin** **beam** held up the **fort**. |
| H | H | **Pile** the **load** into the **hut**. |
| H | H | If you **kick** the **tap** it will **run**. |
| H | H | **Burn** the **heap** of camping **gear**. |
| H | H | Our **guide** drove **far** to get to the **beach**. |
| H | H | **Shut** the large **case** if you need to **hide**. |
| H | H | He **paid** me to **get** his **fan**. |
| H | H | It was **late** when I **lit** the **coal**. |
| L | L | The **soot**-covered **cart** rolled down the **ramp**. |
| L | L | She was **loath** to **jog** through the **slum**. |
| L | L | If you **gawk** at the **mime** he will **wink**. |
| L | L | The **sting** of the **jab** made him **retch**. |
| L | L | **Nuzzle** the **kitten** with the plastic **nipple**. |
| L | L | Don't **spook** the birds that **roost** in the **loft**. |
| L | L | He **knelt** down to **bind** the **yoke**. |
| L | L | **Pierce** the **scab** to draw out the **venom**. |
| L | L | You can't **snooze** as we **lunge** and **swerve**. |
| L | L | **Yawn** as you **fib** about your **badge**. |
| L | L | The **lark** **bled** when hit with the metal **wedge**. |
| L | L | **Prop** up the **trash** to keep in the **sludge**. |
| L | L | The **sleek** fur **pelt** lay where it was **slung**. |
| L | L | The **carp** will **spawn** in a **groove**. |
| L | L | If you **sneak**, you can **scan** the **quiz**. |
| L | L | She will **fret** and **fume** when she wants **fudge**. |
| L | L | **Heckle** the one-**toothed** man to raise his **pulse**. |
| L | L | **Heave** some **moss** onto the **hive**. |
| L | H | The **moat** around the **shack** reached his **shin**. |
| L | H | His **chum** likes to **sip** bottled **pop**. |
| L | H | The **rogue** likes to **hoot** and **jeer**. |
| L | H | **Swat** the **ram** if it starts to **lag**. |
| L | H | **Slap** the **mat** to find the lost **bead**. |
| L | H | Use a wooden **hoop** to **weave** a **shawl**. |
| L | H | I like to **sip** **pop** while I **dine**. |
| L | H | Put a **nail** in the **rim** of the **loom**. |
| L | H | The **lamb** likes to **roam** around the **moat**. |
| L | H | The **bum** stole the **loot** from the **mall**. |
| L | H | The **rash** from the **weed** made her **moan**. |
| L | H | The **lair** will **reek** if you kill the **ram**. |
| L | H | Your **pet** horse likes to **roam** near the **moat**. |
| L | H | The **ram** wants to **rip** the **mat**. |
| L | H | **Hurl** a rotten **peach** at the **witch**. |
| L | H | He **lied** that a **rat** was in the **den**. |
| L | H | I will **pout** if you **maim** my **shin**. |
| L | H | **Soak** the **mum** to get rid of the **bug**. |

**Key Word Properties**

Mean lexical frequency values and mean number of neighbors for key words in the VAST sentences used in the current study.

| Sentence Type | Mean Lexical Frequency of Key Words | Mean number of neighbors of key words |
| --- | --- | --- |
| HL | 97.431 | 14.02 |
| HH | 202.721 | 30.75 |
| LL | 12.732 | 11.902 |
| LH | 11.177 | 31.91 |

*Following Bell and Wilson (2001), lexical frequency values are based on norms from Kučera and Francis (1969) and number of neighbors are defined as number of words that differ from the target keyword by the addition, deletion, or substitution of a single phoneme. Sentence type refers to overall word frequency and neighborhood density values of each key word: high lexical frequency and low neighborhood density (HL); high lexical frequency and high neighborhood density (HH); low lexical frequency and low neighborhood density (LL); and low lexical frequency and high neighborhood density (LH).*
